# Effect of chlorhexidine chip as an adjunct in non-surgical management of periodontal pockets: a meta-analysis

**DOI:** 10.1186/s12903-020-01247-8

**Published:** 2020-09-21

**Authors:** Lili Ma, Xiuchun Diao

**Affiliations:** 1 Department of stomatology, Zaozhuang Maternal and Child Health Hospital, Zaozhuang, 277100 Shandong China; 2 Department of stomatology, Zaozhuang Hospital of Traditional Chinese Medicine, 2666 Taihangshan Road, Xuecheng District, Zaozhuang, Shandong 277000 P.R. China

**Keywords:** Chlorhexidine Chip, Chronic periodontitis, Scaling and root Planing, Periodontal pockets

## Abstract

**Background:**

The aim of this meta-analysis was to evaluate the difference in treatment outcomes between sub-gingival placement of chlorhexidine chip (CHX chip) in adjunct to scaling and root planing (SRP) and SRP alone for the management of periodontal pockets in patients suffering from chronic periodontitis.

**Methods:**

We searched the MEDLINE (PubMed), SCOPUS and CENTRAL databases and identified 15 randomized clinical trials published within the last decade (2007–2019): 9 with split-mouth design and 6 with parallel study design. We extracted data and performed both qualitative and quantitative syntheses. The primary outcomes assessed were gain in clinical attachment level (CAL), reduction in probing pocket depth (PPD), improvement in gingival inflammation, and results of microbiological assays.

**Results:**

We used meta-analysis plots to assess all the clinical outcomes. The mean differences in PPD reductions at 1 month (MD 0.63), 3 months (MD 0.69), and 6 months (MD 0.75); and the CAL gains at 1 month (MD 0.54), 3 months (MD 0.64), and 6 months (MD 0.68) showed more favorable responses in sites treated with the CHX chip as an adjuvant to SRP, than in sites treated with SRP alone.

**Conclusion:**

SRP with adjunctive CHX chips showed better clinical outcomes than SRP alone for the management of periodontal pockets in patients with chronic periodontitis.

## Background

Periodontal disease is characterized by inflammation of tooth supporting structures, and is primarily caused by the presence of dental plaque and calculus [1]. The irreversible periodontium damage caused by noxious substances produced by the plaque micro-flora and the inevitable host response by cytokine release lead to disease progression during chronic periodontitis [2]. The goal of periodontal treatment is to render the tooth surface free from dental plaque and calculus, thereby reducing or eradicating periodontal pathogens and allowing periodontal tissues to restore their health [3]. Treatment is primarily achieved by nonsurgical methods of periodontal therapy [4–7].

Scaling and root planing (SRP) is an effective nonsurgical periodontal therapy for chronic periodontitis at its early stages [8]. However, the operator’s accessibility to clean deep periodontal pockets remains limited in cases of furcation, multi-rooted teeth, developmental grooves, root concavities, mal-occlusion, and inter-proximal areas. This limitation to proper instrumentation in inaccessible areas compromises the effectiveness of SRP [9]. This has led to the use of antimicrobials as an adjunct to SRP, assuming that such agents would aid to treat the dysbiosis in these inaccessible areas and help prevent microbial colonization to promote clinical improvements. Studies have shown that local as well as systemic anti-microbial agents have a beneficial effect on non-surgical periodontal therapy [10, 11]. Locally administered antimicrobial agents are, however, preferred since they are associated with less systemic side-effects than systemic antimicrobials. A number of local antimicrobial agents have been advocated as adjuvants for management of periodontal diseases [12]. But, attempts to administer these agents locally inside the periodontal pocket were limited by lack of retention and inability to achieve adequate inhibitory concentrations in the gingival crevicular fluid [13, 14].

Chlorhexidine (CHX) is a bis-biguanide molecule made up of two (p-chlorophenyl) guanide units linked by a hexamethylene bridge. It is a potent anti-infective and antibacterial mouth-rinse agent used as a prophylactic and therapeutic measure against periodontal disease [15]. Despite having a high substantivity as compared to other anti-microbial agents, the subgingival availability of CHX is limited and questionable when used as mouth-wash [16].

Local CHX delivery has shown clinical benefits when compared to the use of mouth rinse [17]. A CHX chip is a resorbable chip with 2.5 mg of CHX embedded in a cross-linked hydrolyzed gelatin matrix. When sub-gingivally delivered into deep periodontal pockets, the chip releases a controlled amount of CHX with simultaneous biodegradation over a 1 week period providing a CHX concentration lower than 125 mg/ml to the gingival sulcular fluid [18].

A systematic review on adjunctive use of the CHX chip with SRP failed to provide conclusions in terms of the chip’s treatment effectiveness due to inadequate numbers of studies available at the time [19]. Six studies included in the systematic review were of low quality, had high heterogeneity and lacked information on allocation concealment and follow-ups. With publication of several studies since then, there is a need for updated evidence on the subject. Therefore, the purpose of this systematic review and meta-analysis was to analyze the literature to assess the efficacy of sub-gingival CHX chip when used as adjunct with SRP for non-surgical treatment of periodontal pockets in patients with chronic periodontitis.

## Methods

We based our review on the preferred reporting items for systematic review and meta-analysis (PRISMA) guidelines, and performed both qualitative and quantitative syntheses to evaluate the treatment outcomes.

### Research question

What are the treatment outcomes of SRP with and without adjunct subgingival CHX chip for managing periodontal pockets in patients with chronic periodontitis?

- *Patient/Population:* Patients with chronic periodontitis and periodontal pockets > 4 mm.
- *Intervention:* Subgingival delivery of CHX chip in addition to SRP.
- *Comparison:* Patients treated with SRP alone.
- *Outcomes:* Clinical and Microbiological outcomes.

### Search strategy

We performed a systematic digitalized search in electronic databases like MEDLINE (PubMed), Scopus, and CENTRAL (the Cochrane Central Register of Controlled Trials) using relevant keywords and strategically employed terms like ‘AND’, ‘OR’, and ‘NOT’.

The strategy employed for the electronic search was as follows: “(chlorhexidine chip, periocol, periochip, OR “chlorhexidine“, controlled release devices, subgingival delivery) or (“biguanides“ AND “non-surgical periodontal therapy“ OR “periodontal pockets”, “chronic periodontitis“, or periodon* OR “periodontal disease/therapy”)”.

We also carried out a manual search in recent issues of dental journals: Clinical Oral Investigations, European Journal of Oral Sciences, Journal of Periodontics and Restorative Dentistry, Journal of Clinical Periodontology, Journal of Dental Research, Journal of Dentistry, Journal of Periodontal Research, Journal of Periodontal and Implant Science, and Journal of Periodontology.

In addition, we screened the bibliography column of relevant clinical reports and reviews for any additional eligible clinical studies.

### Selection criteria

The following criteria’s were used to select potential eligible studies from the list of studies identified through our electronic and manual searches:

- Randomized clinical trials (RCTs)
- Studies employing CHX chips as an adjunct to SRP in an experimental group with comparisons to a control group with SRP alone during management of periodontal pockets > 4 mm in patients suffering from chronic periodontitis
- Participants included in the studies were free from systemic diseases;
- Studies with participants followed for at least 1 month
- Studies published within 1st January 2006 to 1st January 2020.

### Study selection

We compiled the studies retrieved from the electronic database searches into a citation manager software (EndNote v7.0, Clarivate Analytics, USA) to remove duplicates. After that, two independent reviewers screened all the studies based on titles and abstracts. The potential eligible studies were subjected to full text assessment and tagged under included studies if they satisfied the selection criteria.

### Data extraction

We used an Excel Spreadsheet (Microsoft, Radmond, WA, USA) to retrieve relevant detailed information from the included studies for qualitative synthesis. Two independent reviewers extracted all data from the included studies separately to eliminate errors in extraction of variables and outcomes. We contacted authors of papers missing–or with incomplete or unclear– information by telephone or email to obtain the complete details or clarify information.

### Outcomes

Our outcomes included the probing pocket depth (PPD), clinical attachment level (CAL), gingival inflammation scores, and microbiological findings recorded at all follow-up periods.

### Data synthesis

We analyzed the extracted data both quantitative and qualitatively, and tabulated the qualitative data and the demographics details from all the included studies. A meta-analysis was conducted when at least two studies assessed the same clinical outcome at comparable follow-up periods. Continuous data was pooled using the mean differences (MD) with 95% confidence intervals (CI). The statistical analysis units of measurement for each outcome were tooth sites, not single patients. We used the Review Manager 5.3 software (RevMan 5.3, Version 5.3.5 Copenhagen: The Nordic Cochrane Centre, The Cochrane Collaboration, 2014.) for the meta-analysis according to the fixed or random effects models, as appropriate. We used a fixed effects meta-analysis when the heterogeneity was small (*I*^*2*^ *< 60%, P > 0.05*), and a random-effects model analysis when the heterogeneity was large (*I*^*2*^ *> 60%, P < 0.05*).

### Risk of bias assessment

The risk of bias assessment was carried out according to guidelines in the Cochrane Handbook of Systematic Reviews of Interventions using Revman 5.3 software. We carefully assigned bias pertaining to randomization process and allocation concealment, blinding of participants, personnel or assessor, and any incomplete or selective outcome data reporting to assess risks of bias for all the included studies. Two reviewers independently judged the assessments and consulted a third reviewer in cases of discrepancies or doubt to arrive at a consensus. The individual assessments of bias were judged as low in case of valid information, unclear in case of lack of clarity, and high in case of missing or invalid information. The reviewers further graded the studies based on their bias assessment scores (low risk studies had low scores on all assessments, medium risk studies had one or more unclear bias assessment scores; and high risk studies had high scores).

## Results

**Fig. 1:**
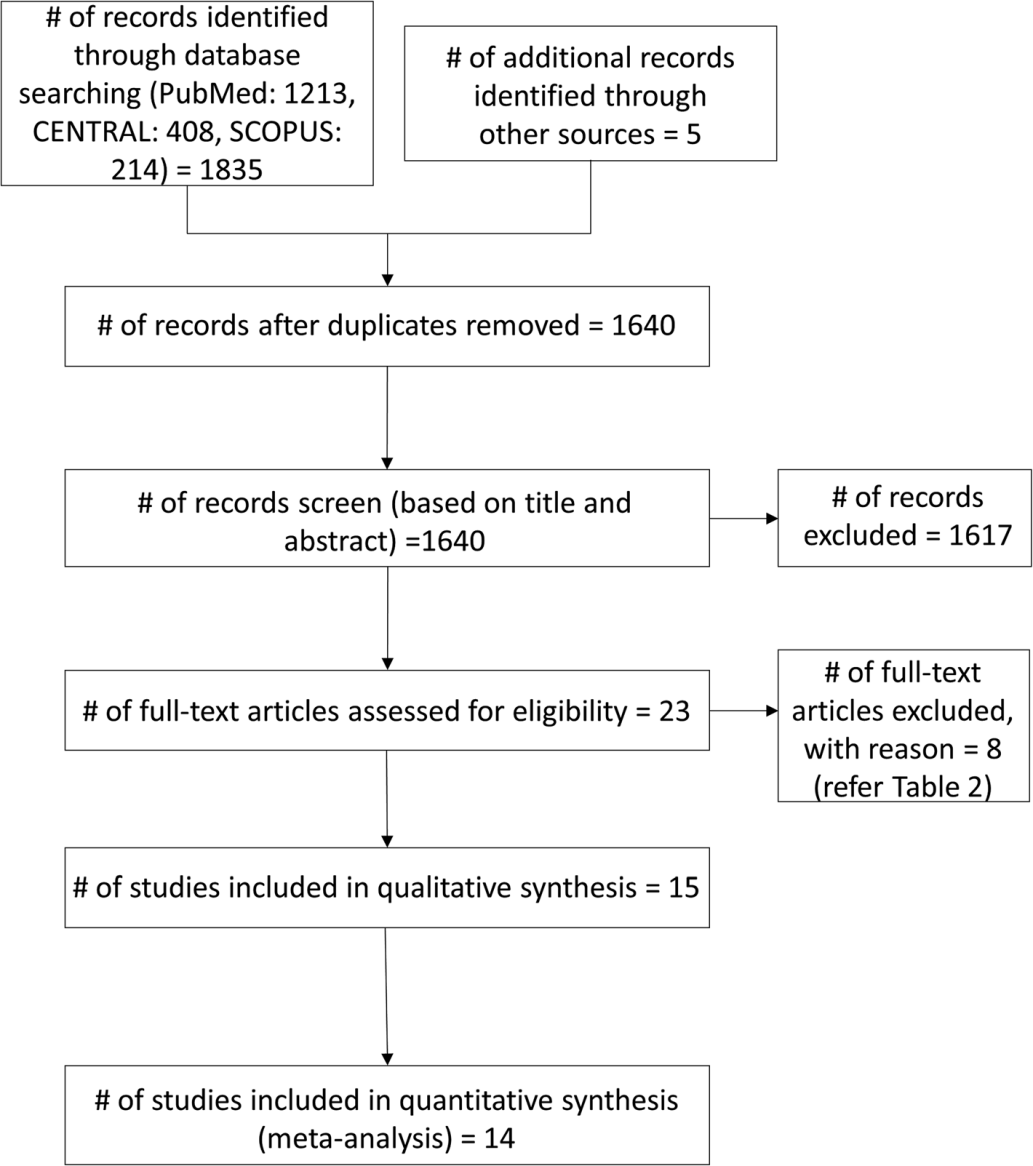
PRISMA flow chart of study selection process

The systematic selection process of eligible studies is provided in the PRISMA flow chart (Fig. 1). A total of 1640 unique records were identified of which 15 studies were included. Details of included studies are presented in Table 1. Table 2 lists the reasons for exclusion of studies after full text assessments.

**Table 1:** General Characteristics of studies included

Study	Author & year	Study design	Age range	Gender (M/F)	Sample size	Groups	CHX chip company/make	Outcomes	Study duration
1	*Sahu* et al. *2019* [20]	RCT (SM)	25–55	NM	20 patients (40 sites)	A. SRP alone (20 sites) B. SRP plus CHX chip (20 sites)	PERIOCOL-CG™ (2.5 mg CHX from a 20% CHX solution in fish collagen membrane)	PI,GI,SBI,PPD, RAL	3 months (0,1,3)
2	*Singh* et al. *2018* [21]	RCT (SM)	35–55	15/5	20 patients (40 sites)	A. SRP alone (20 sites) B. SRP plus CHX chip (20 sites)	NM	PI, m-BI, PPD, CAL, BANA	3 months (0,1,3)
3	*Divya* et al. *2018* [22]	RCT (P)	NM	NM	122 sites	A. SRP alone (61 sites) B. SRP plus CHX chip (61 sites)	PERIOCOL-CG™ (2.5 mg CHX from a 20% CHX solution in fish collagen membrane)	GI, PPD,CAL	9 months (0,1,3,6,9)
4	*Singh* et al. *2017* [23]	RCT (SM)	30–50	22/18	40 patients (120 sites)	A. SRP alone (40 sites) B. SRP plus CHX chip (40 sites) C. SRP plus Turmeric Chip (40 sites)	PERIOCOL-CG™ (2.5 mg CHX from a 20% CHX solution in fish collagen membrane)	PI, GI, PPD, RAL	3 months (0,1,3)
5	*Lecic* et al. *2016* [24]	RCT (SM)	21–52	8/7	15 patients (120 sites)	A.SRP alone (60 sites) B. SRP pus CHX gel (20 sites) C.SRP plus CHX irrigation (20 sites) D. SRP plus CHX chip (20 sites)	Perio Chip®, Perioproducts, Jerusalem, Israel	PI, BOP, PPD, CAL	3 months (0,1,3)
6	*John* et al.*, 2015* [25]	RCT (SM)	35–56	11/9	20 patients (40 sites)	A. SRP alone (20 sites) B. SRP plus CHX chip (20 sites)	2.5 mg CHX from a 20% CHX solution in fish collagen membrane	PI, GI, PPD, CAL	3 months (0, 11 days, 11 weeks)
7	*Pattnaik* et al.*, 2015* [26]	RCT (SM)	29–54	9/11	20 patients (40 sites)	A. SRP alone (10 sites) B. SRP plus CHX chip (10 sites)	PERIOCOL-CG™ (2.5 mg CHX from a 20% CHX solution in fish collagen membrane)	PD, CAL, GI, Bacterial Count	3 months (0, 1, 3)
8	*Kumar* et al.*, 2014* [27]	RCT (P)	20–65	15/15	30 patients (30 sites)	A. SRP alone (10 sites) B. SRP plus CHX chip (10 sites) C. CHX chip alone (10 sites)	PERIOCOL-CG™ (2.5 mg CHX from a 20% CHX solution in fish collagen membrane)	GI, PPD, CAL, BANA	0, 1, 3 m
9	*Medaiah* et al. *2014* [28]	RCT (P)	35–55	6/9	15 patients (45 sites)	A. SRP alone (15 sites) B. SRP plus CHX chip (15 sites) C. CHX chip alone (15 sites)	Perio Chip®, Perioproducts, Jerusalem, Israel	PI, GI, BOP, PD, CAL	3 months (0,1,3)
10	*Pai* et al. *2013* [17]	RCT (P)	35–55	7/8	15 patients (45 sites)	A. SRP alone (15 sites) B. SRP plus CHX Varnish (15 sites) C. SRP plus CHX chip (15 sites)	NM	PI, BOP, SBI, PPD, CAL	3 months (0,1,3)
11	*Puri* et al. *2013* [29]	RCT (SM)	30–50	8/7	15 patients (30 sites)	A. SRP alone (15 sites) B. SRP plus CHX chip (15 sites)	Perio Chip®, Perioproducts, Jerusalem, Israel	GI, PI, PPD, CAL, TCC	3 months (0,1,3)
12	*Grover* et al.*, 2011* [30]	RCT (P)	30–65	28/12	40 patients (40 sites)	A. SRP alone (20 sites) B. SRP plus CHX chip (20 sites)	PERIOCOL-CG™ (2.5 mg CHX from a 20% CHX solution in fish collagen membrane)	PPD, CAL, BI and Radiological parameters (bone gain)	3 months (7th day, 1, 2, 3)
13	*Sakellari* et al.*, 2010* [31]	RCT (P)	35–75	25/25	50 patients (50 sites)	A. SRP alone (25 sites) B. SRP plus CHX chip (25 sites)	Perio Chip®, Perioproducts, Jerusalem, Israel	PPD, CAL, BOP, Bacterial Count	6 months (0, 3 weeks, 3,6)
14	*Paolantonio* et al.*, 2008* [32]	RCT (SM)	33–65	34/82	116 patients (232 sites)	A. SRP alone (116 sites) B. SRP plus CHX chip (116 sites)	Perio Chip®, Perioproducts, Jerusalem, Israel	PPD, RAL, BOP, Bacterial Count	6 months (0, 3, 6)
15	*Paolantonio* et al.*, 2008* [33]	RCT (SM)	31–63	33/49	82 patients (164 sites)	A. SRP alone (82 sites) B. SRP plus CHX chip (82 sites)	Perio Chip®, Perioproducts, Jerusalem, Israel	PPD, RAL, BOP, GCF- ALP	6 months (0, 3, 6)

Legend: *RCT* Randomized controlled trial, *SM* Split-mouth, *P* Parallel, *SRP* Scaling and root planing, *CHX* Chlorhexidine, *PI* Plaque index, *GI* Gingival index, *BI* Bleeding index, *SBI* Sulcus bleeding index, *PPD* Probing pocket depth, *CAL* Clinical attachment level, *RAL* Relative attachment level, *BOP* Bleeding on probing, *TCC* Total colony count, *BANA* N-benzoyl D, L-arginine-2-naphthylamide test kit, *GCF* Gingival crevicular fluid, *ALP* Alkaline phosphatase

**Table 2:** Reasons for excluding studies

Study	Author & year	Reason for exclusion
1	Konugati et al. 2016 [34]	Positive control (Flurbiprofen)
2	Jhinger et al. 2015 [35]	Not a randomized clinical trial
3	Singh et al. 2014 [36]	Low quality RCT (Randomization not clear)
4	Kondreddy et al. 2012 [37]	Comparative study
5	Matchei et al. 2011 [38]	Positive control (Flurbiprofen)
6	Gonzales et al. 2011 [39]	Use of placebo as control
7	Tara Paul et al. 2010 [40]	Comparison with surgical treatment
8	Kasaj et al. 2007 [41]	Supportive periodontal therapy

Out of 15 included reports, nine were split mouth studies, and the other six were of parallel design. The included studies involved a total of 620 patients with 998 treatment sites randomly divided into SRP alone (509 sites) and SRP plus CHX chip (489 sites) groups. The sample size per arm varied from a minimum of 30 sites to a maximum of 232 sites. The CHX chips used in the studies had 2.5 mg of CHX (20% CHX embedded in a collagen matrix) under the brand name of PERIOCOL –CG (Eucare Pharmaceuticals, India) or PERIOCHIP (Perioproducts, Jerusalem, Israel). The follow-up duration among the studies varied from a minimum of 1 month to a maximum of 9 months, with maximum outcomes recorded at the end of 1 and 3 months.

**Fig. 2:**
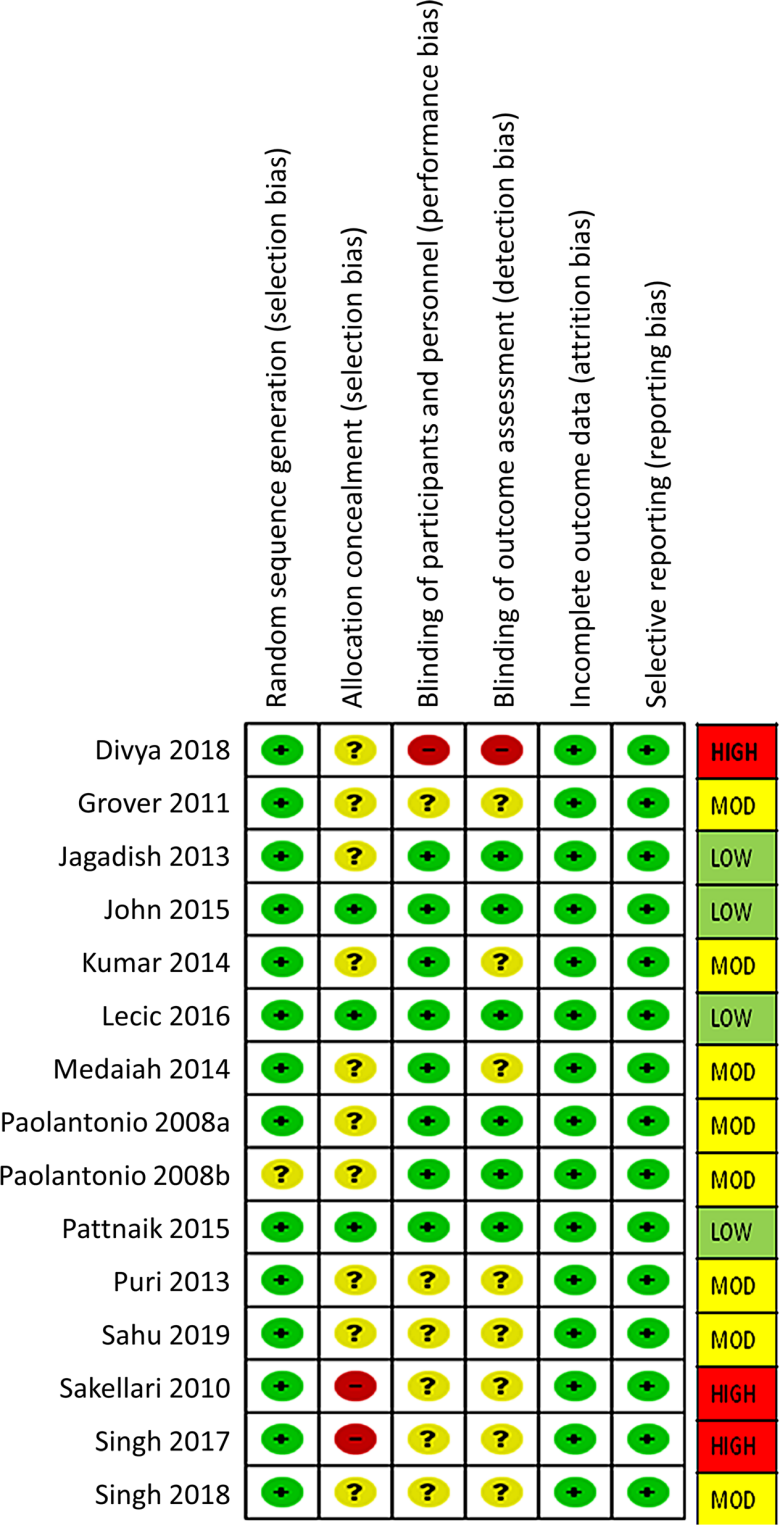
Risk of Bias (RoB) assessment for included studies

We found eight studies [20, 21, 27–30, 32, 33] with moderate and four [17, 24–26] with low risk of bias. Three of the trials [22, 23, 31] had high risks of bias due to lack of information or inappropriateness regarding allocation concealment and blinding of participants, personnel, or outcome assessors (Fig. 2).

We carried out quantitative analyses to compare outcomes between the groups based on gain in CAL, reduction in PPD, and improvement in gingival inflammation scores. We used data from 14 studies for our meta-analysis plots. We plotted MD between the groups of the 14 included studies into forest plots at all follow-ups. We also performed subgroup analyses according to their study design.

### PPD reductions

#### At 1-month follow-up

**Fig. 3:**
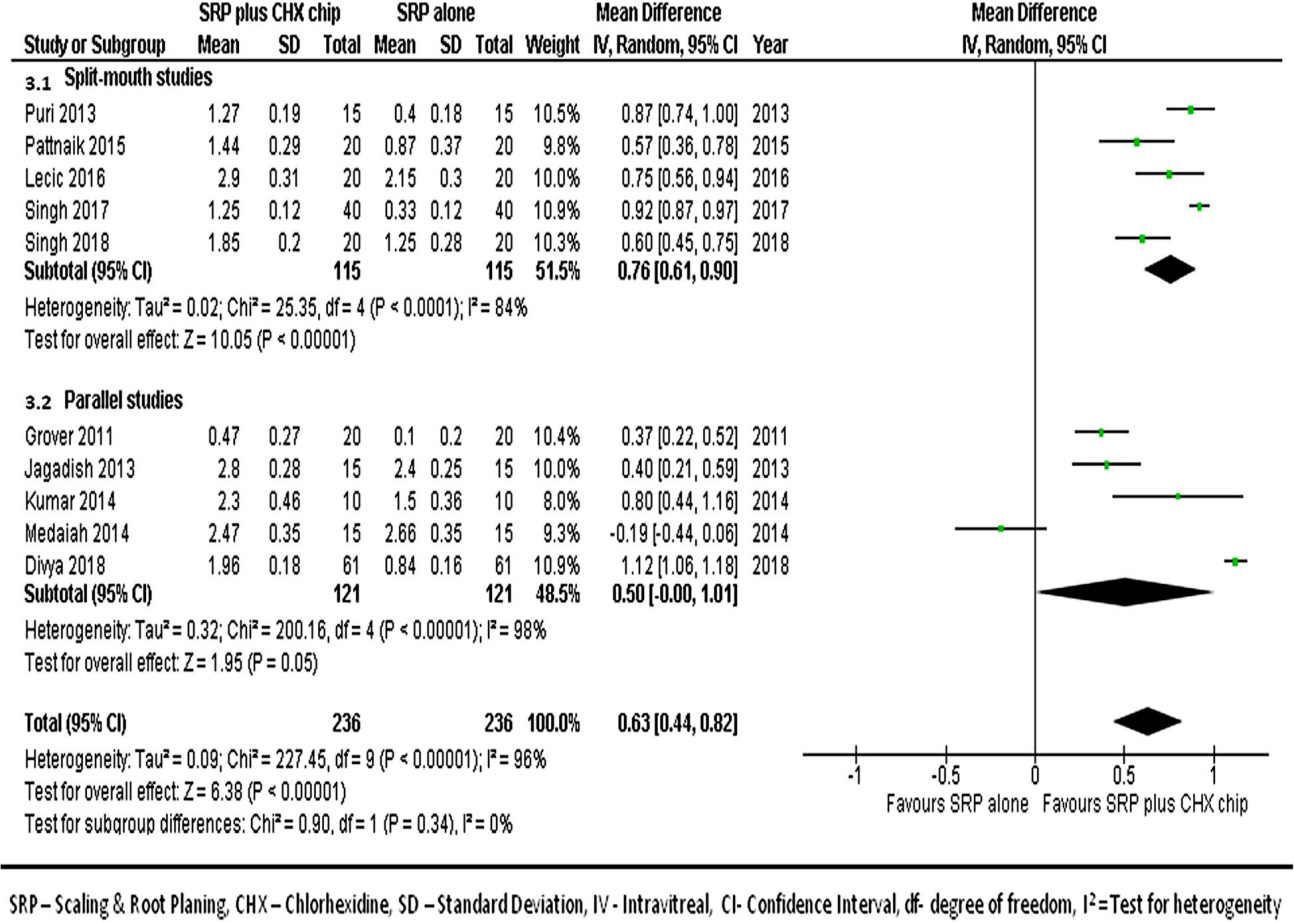
Forest plot showing the mean difference in PPD reduction at 1-month follow-up compared to baselines between SRP + CHX and SRP alone groups

We combined data from 10 studies [17, 21–24, 26–30] to compare the reduction in PPD between the groups at the 1 month follow-ups. Figure 3 shows the forest plot for the mean differences in PPD reduction PPD at 1-month between groups, which suggests the sites treated with SRP and CHX chip had better outcomes than the sites treated with SRP alone (MD, 0.63; 95% CI 0.44–0.82; *p* < 0.001).

#### At 3-month follow-up

**Fig. 4:**
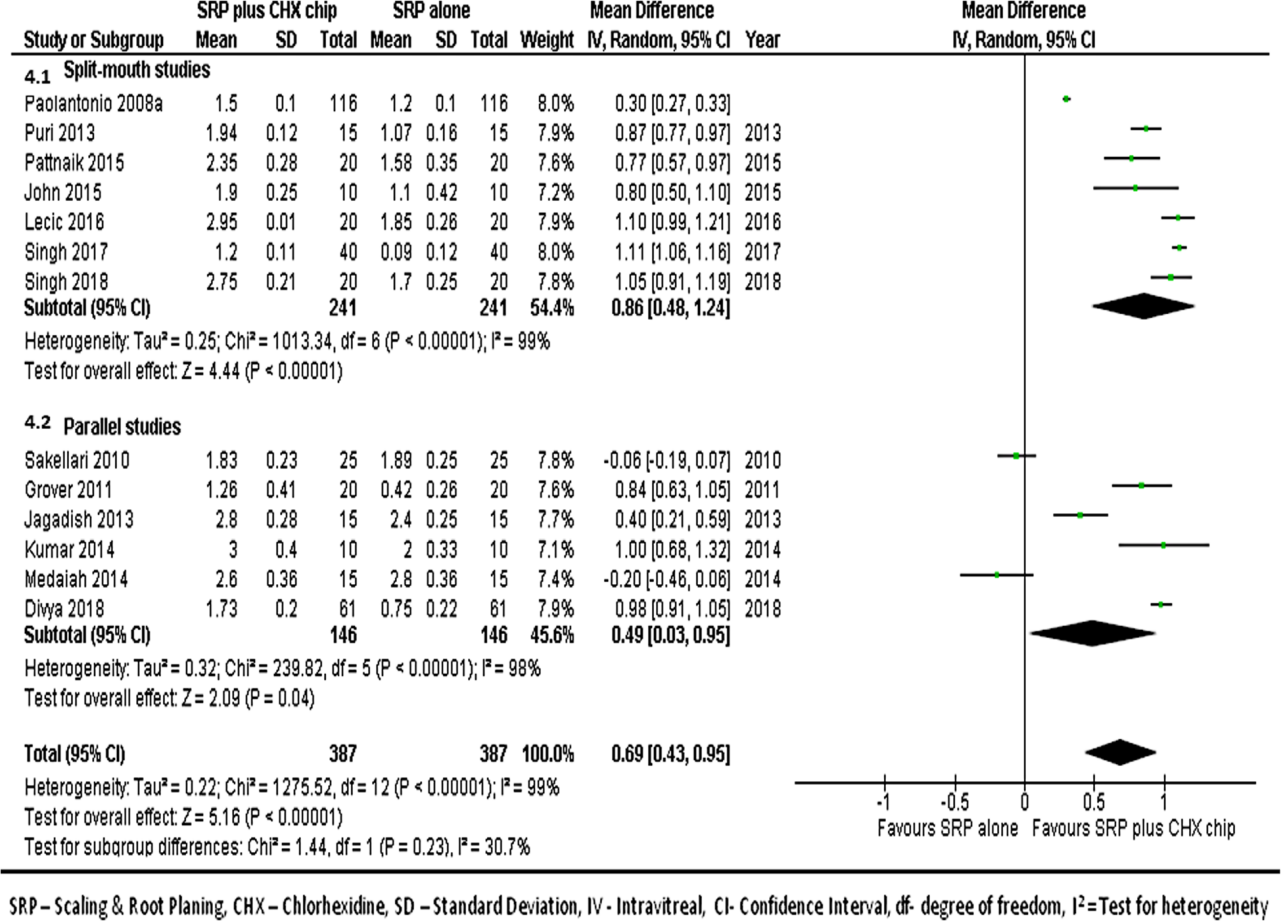
Forest plot showing the mean difference in PPD reductions at 3-month follow-up compared to baselines between SRP + CHX and SRP alone groups

We combined data from 13 studies [17, 21–32] to compare the PPD reduction between the groups at the 3 month follow-ups. Figure 4 shows the forest plot with mean differences in PPD reduction between groups and suggests that the sites treated with SRP and CHX chip had a better response than those treated with SRP alone (MD, 0.69; 95% CI, 0.43–0.95; *p* < 0.001).

#### At 6-month follow-up

**Fig. 5:**
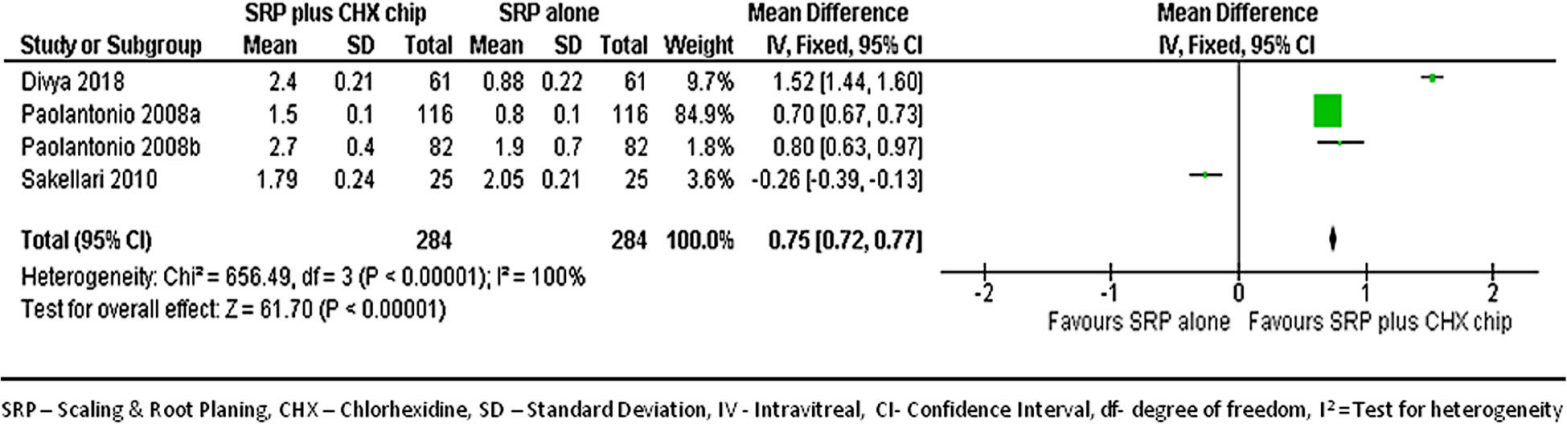
Forest plot showing the mean difference in PPD reductions at 6-month follow-up compared to baselines between SRP + CHX and SRP alone groups

We combined data from only 4 studies [22, 31–33] to compare the PPD reduction between the groups at the 6-month follow-ups. Figure 5 shows the forest plot with mean differences in PPD reduction between groups, and suggests the sites treated with SRP and CHX chip had a better response than those treated with SRP alone (MD, 0.75; 95% CI, 0.72–0.77; *p* < 0.001).

### CAL gains

#### At 1-month follow-up

**Fig. 6:**
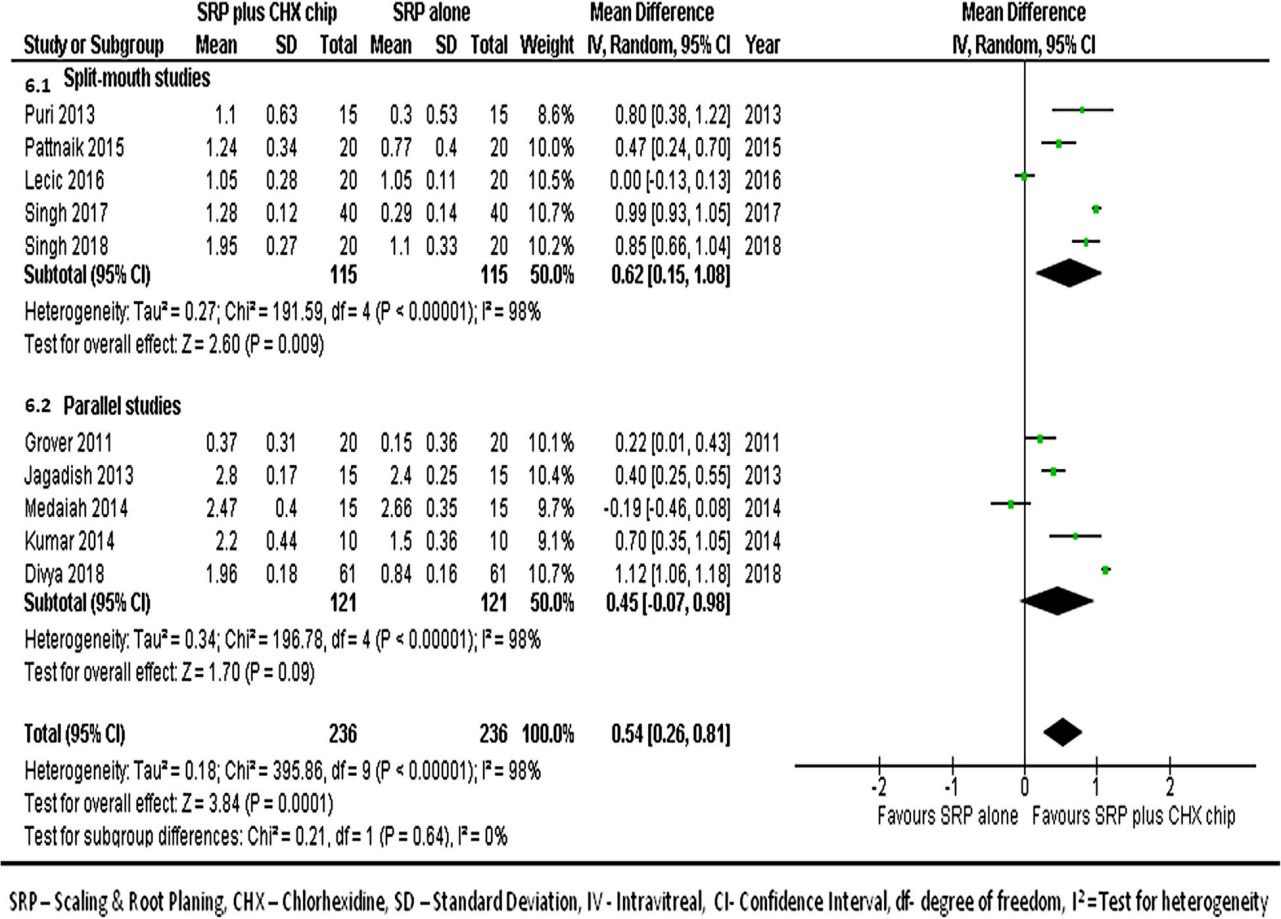
Forest plot showing the mean difference in clinical attachment gains at 1-month follow-up compared to baselines between SRP + CHX and SRP alone groups

We combined data from 10 studies [17, 21–24, 26–30] to compare CAL gains between groups at the 1-month follow-ups. Figure 6 shows the forest plot with mean differences in CAL gains between groups, suggesting that the sites treated with SRP and CHX chip had a better response than those treated with SRP alone (MD, 0.54; 95% CI, 0.26–0.81; *p* < 0.001).

#### At 3-month follow-up

**Fig. 7:**
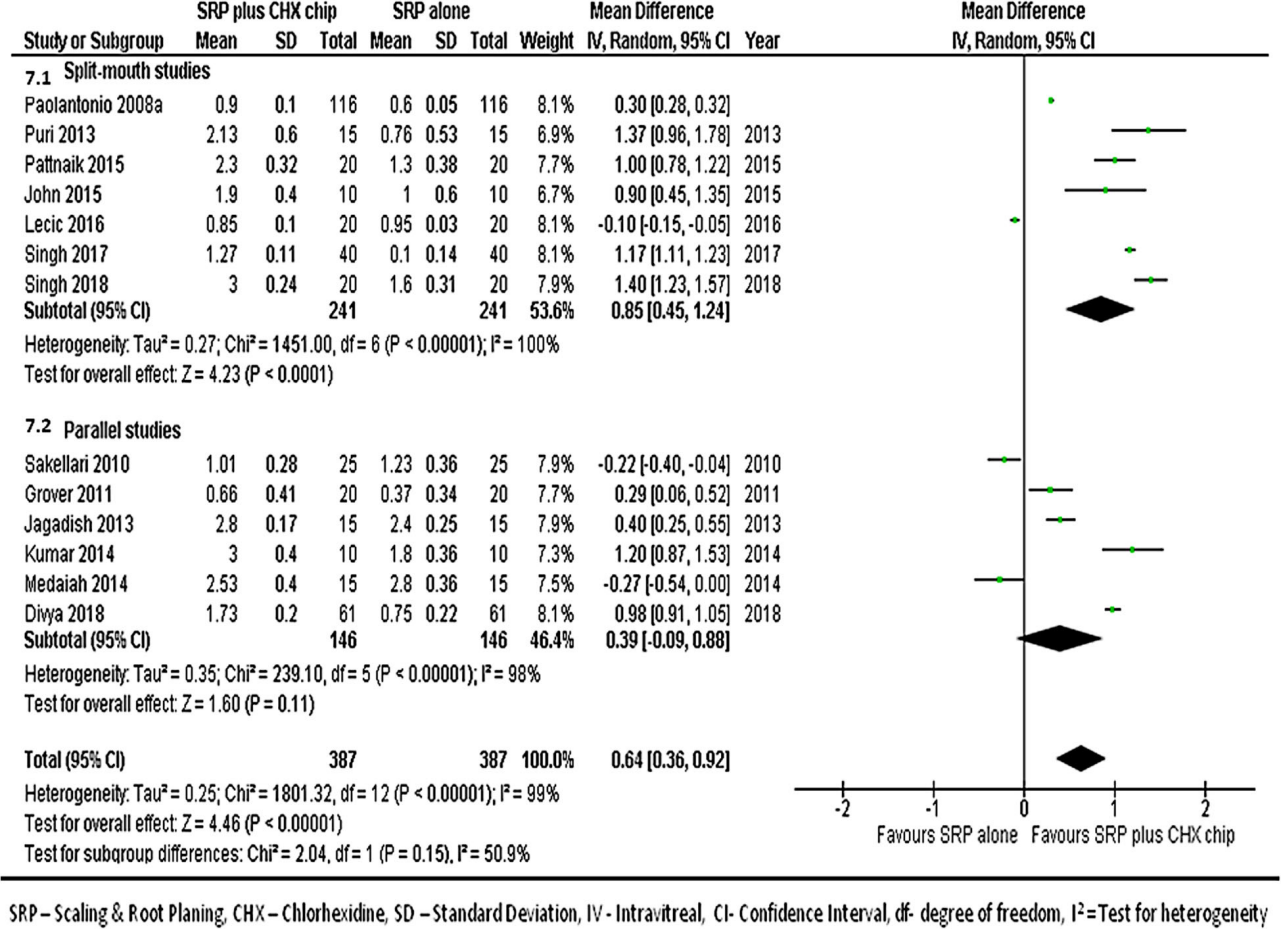
Forest plot showing the mean difference in clinical attachment gains at 3-month follow-up compared to baselines between SRP + CHX and SRP alone groups

The combined data from 13 studies [17, 21–32] to compare the CAL gains between groups at the 3 month follow-ups shows the forest plot with mean differences in CAL gains between groups (Fig. 7), suggesting that sites treated with SRP and CHX chip had a better response than those treated with SRP alone (MD, 0.64; 95% CI, 0.36–0.92; *p* < 0.001).

#### At 6-month follow-up

**Fig. 8:**
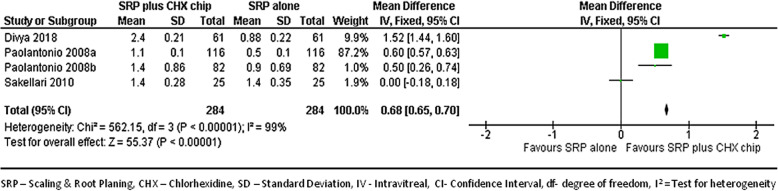
Forest plot showing the mean difference in clinical attachment gain at 6-month follow-up compared to baselines between SRP + CHX and SRP alone groups

We combined data from 4 studies [22, 31–33] to compare the CAL gains between groups at the 6 month follow-ups. Figure 8 shows the forest plot with mean differences in CAL gains between groups, suggesting that the sites treated with SRP and CHX chip had a better response than those treated with SRP alone (MD, 0.68; 95% CI, 0.65–0.70; *p* < 0.001).

### Gingival inflammation improvement

#### At 1-month follow-up

**Fig. 9:**
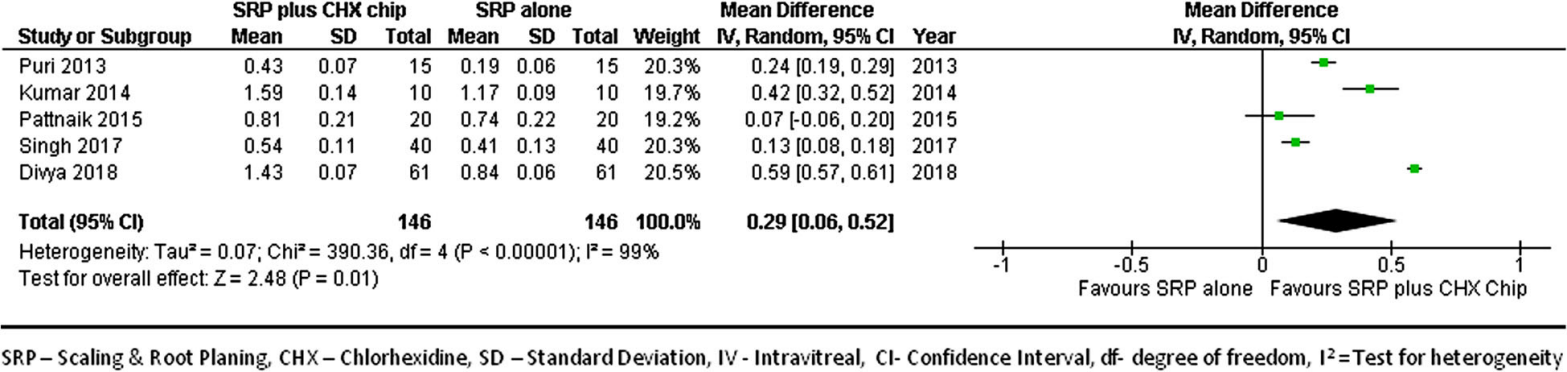
Forest plot showing the mean difference in gingival inflammation reduction at 1-month follow-up compared to baselines between SRP + CHX and SRP alone groups

We combined data from 5 studies [22, 23, 26, 27, 29] to compare gingival inflammation score improvements between groups at 1-month follow-ups. Figure 9 shows the forest plot with mean differences in GI score improvements between groups, suggesting that sites treated with SRP and CHX chip had a better response than those treated with SRP alone (MD, 0.29; 95% CI, 0.06–0.52; *p* < 0.001).

#### At 3-month follow-up

**Fig. 10:**
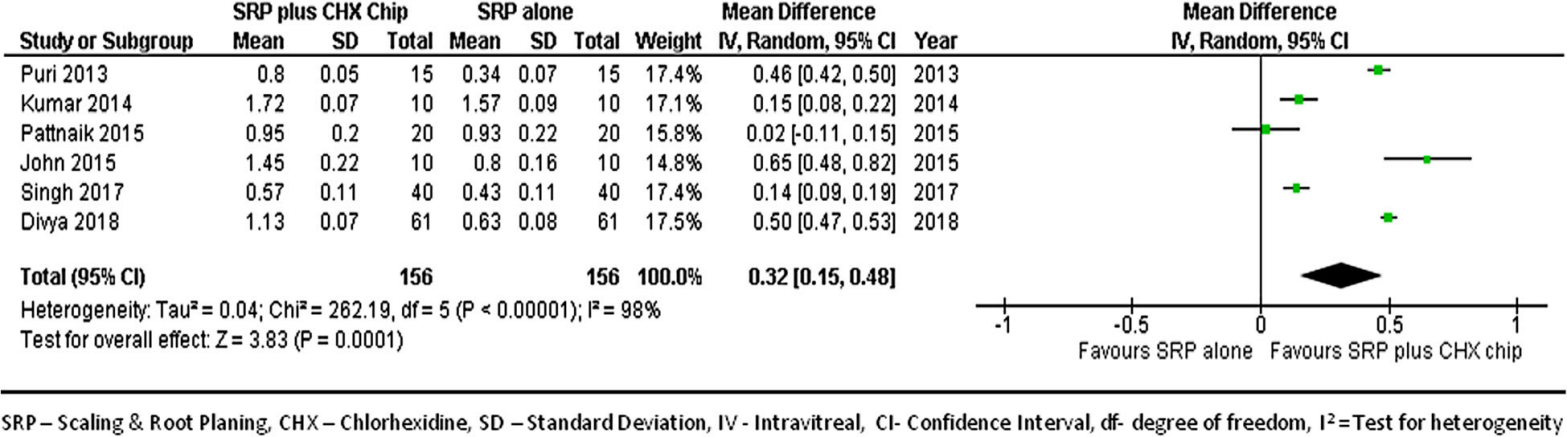
Forest plot showing the mean difference in gingival inflammation reduction at 3-month follow-up compared to baselines between SRP + CHX and SRP alone groups

We combined data from 6 studies [22, 23, 25–27, 29] to compare the GI score improvements between groups at 3-month follow-up. Figure 10 shows the forest plot with mean differences in GI score improvements between groups, suggesting that the sites treated with SRP and CHX chip had a better response than those treated with SRP alone (MD, 0.32; 95% CI, 0.15–0.48; p < 0.001).

#### Microbiological findings

We did not attempt to produce a forest plot to compare microbiological outcomes (such as total colony counts, reduction in periodontal pathogens, or color change in BANA test kit) because of unavailability of more than 2 studies with similar outcome variables.

However, we produced a qualitative synthesis of the included reports evaluating microbiological outcomes. Two studies [21, 27] evaluating %BANA positive sites showed significant reductions in the amount of sites positive for *Treponema denticola*, *Tannerella forsythia,* and *Porphyromonas gingivalis* when treated with SRP plus CHX chip. We also found similar results in other 4 studies evaluating mean periodontal pathogens reductions through quantitative-PCR that showed better outcomes in sites treated with SRP and CHX chip than in those treated with SRP alone [23, 26, 29, 32].

## Discussion

This systematic review and meta-analysis evaluated the effect of the CHX chip as an adjunct treatment to SRP for the management of periodontal pockets in patients with chronic periodontitis. The RCTs included in this meta-analysis were mostly of low and moderate risks of bias. However, three trials had high risks of bias due to lack of allocation concealment or inadequacy in blinding of participants, personnel, or outcome assessors.

CHX has shown promising and effective clinical benefits when used as a mouthwash [15]. However, its effectiveness in deep subgingival areas and inaccessible anatomical contours has remained unclear. Consequently, CHX chips have been devised which allow for ease of placement into the subgingival sites and provide sustained release of CHX over a period of time. The efficacy of CHX chips against periodontal pathogens has been a subject of research, however, the results have been conflicting [42, 43]. An in-vitro study has shown that *Porphyromonas gingivalis* can inactivate the CHX molecule by releasing vesicles that surround the microorganism’s capsule, thereby protecting it and also other micro-organisms from the antibacterial effects of CHX [33]. On the other hand, CHX has been reported to be a potent anti-bacterial agent that inhibits microbial proteases released from potent periodontopathogens [44]. Thus, whether the anti-microbial effect of CHX chips results in clinical benefits when placed into deep periodontal pockets needs to be thoroughly investigated.

In one of the earliest systematic reviews on the subject, Cosyn et al. in 2006 reviewed 5 RCTs studying the adjunctive use of CHX chip with SRP. However, due to the limited and conflicting data from the included studies, the authors failed to derive strong conclusions and suggested the need for more RCTs to confirm the beneficial effect of CHX chips over conventional nonsurgical periodontal treatment [19]. That review also concluded that SRP is a prerequisite for any chemotherapy or local drug delivery agent such as the CHX chip, limiting the beneficial effects of CHX chips used alone without root planing [19].

In the current study, we included RCTs only from 2007 to 2019 with an intention to provide a review of only the latest evidence on the topic published after the Cosyn et al. [19] review. The primary outcomes assessed in the studies of this review included PPD reduction, CAL gain, GI improvement, and bleeding on probing (BOP). The assessment of BOP is a clear indicator of pocket activity. But, as only a few studies assessed BOP with heterogenous data collection (some assessing presence of BOP alone, and others percentage of BOP sites), a meta-analysis could not be performed.

PPD is a commonly used diagnostic tool for assessing destruction of periodontal structures and PPD along with CAL are important clinical indicators for both diagnosis of periodontal disease and monitoring the of success of treatment [24]. In our analysis, we found a statistically significant difference in PPD reductions between the study groups at 1 month (MD 0.63), 3 months (MD 0.69), and 6 months (MD 0.75) with results favoring sites treated with CHX chip and SRP. The improvement in the PPD with CHX and SRP was seen in all studies except for Medaiah et al. [28] and Sakellari et al. [31]. Our meta-analysis also demonstrated significant difference in CAL between the study groups at 1 month (MD 0.54), 3 months (MD 0.64), and 6 months (MD 0.68) with results favoring sites treated with CHX chip plus SRP. These findings concur with previous reviews on the topic. Smiley et al. [45] in a systematic review and meta-analysis published in 2015 have analyzed the efficacy of local adjuncts in combination with SRP. The inclusion criteria of this review were limited to studies with a minimum 6 months follow-up with CAL as primary outcome. After an analysis 6 RCTs, the authors reported a statistical significant increase of CAL (MD 0.4, 95% CI 0.24–0.56) with the use of CHX chip as an adjunct to SRP as compared to SRP alone. However, no meta-analysis was performed for PPD in their study.

Another review by Matesanz Perez et al. [46] published in 2013 has investigated the effect of local antimicrobials as an adjunct to subgingival debridement in the management of chronic periodontitis. In a sub-group analysis, data from 9 trials assessing the efficacy of CHX chips as an adjunct to SRP was pooled [46]. The authors reported no statistical significant difference in PPD between the study groups at short-term follow-up (< 6 months) (*p* = 0.321), but reported significantly better outcomes with CHX chips after a follow-up of 6–12 months) (*p* < 0.001). Similar findings were also recorded for change in CAL. The included studies also demonstrated significant heterogeneity in the PPD and CAL assessments [46]. Similarly, our meta-analysis also showed very high heterogeneity among the included studies in terms of the clinical parameters studied. This may be attributed to several factors like the variation in the study populations, disease severity, quality of SRP, operator’s experience, etc. Such heterogeneity in the meta-analysis may cause over- or under-estimation of the treatment effect of the CHX chips, limiting the results of our systematic review.

While discussion the results of CHX as an adjunct to non-surgical therapy, it is important to distinguish the effects of various modes of delivery of CHX. CHX chip has proved itself to be more effective as compared to other forms like CHX irrigation or gel [24].. Due to the slow degradation of the chip, CHX is released in a gradual and sustained manner for a longer period of time. In comparison, the gel form, though delivered locally in to the periodontal pocket, does not provide for sustained release of CHX. Similarly, CHX irrigation provides only a short-term effect due to the drugs substantivity of 12 h. Furthermore, the continuous flow of gingival crevicular fluid in the periodontal pocket hinders the retention of CHX solution.

One of the major limitations of this study is our failure to perform a meta-analysis for the assessment of periodontal pathogen colony count reductions and BOP due to unavailability of adequate trials assessing these variables. Other drawbacks include a lack of descriptions of SRP instruments (manual or ultrasonic scalers) as well as the duration of the instrumentation and operator variations. Another source of heterogeneity among the included studies is due to effects of multiple applications of CHX chips. One of the studies mentioned the use of second CHX chips to improve outcomes [22]. However, the placement of the second chip was made at the end of 6 months and would not have affected our meta-analysis results [22]. The results of the mentioned study were significantly better for sites with SRP and CHX chips than for sites with SRP alone, even after 9 months of follow-up, suggesting that multiple CHX chip applications could be of added benefit for long term effects [22]. Lastly, the quality of overall evidence was moderate as majority of the included studies did not provide details of allocation concealment, blinding of personnel and blinding of outcome assessors. This may have introduced bias in the overall results of our analysis.

The results of this review seem to indicate that the CHX chip may serve as a useful adjuvant to non-surgical periodontal therapy. However, the combination of CHX and SRP cannot be considered as a gold standard treatment. Due to better outcomes with CHX, the need for surgical therapy for treating periodontal pockets may be minimized. Clinicians should assess the need for surgery on a case-to-case basis while also considering the use of CHX chip with non-surgical therapy. The clinical outcomes with CHX chip as an adjunct to SRP also depend upon the baseline PPD, the adequacy of SRP as well as on the patient’s compliance, systemic disease status, and smoking habit. These criteria’s should be considered while recommending the use of CHX chips to treat periodontal pockets.

## Conclusion

Within the study limitations, our results indicate that clinical outcomes may be significantly improved in patients undergoing non-surgical therapy for periodontal pockets with the adjunctive use of CHX chip after SRP as compared to SRP alone. The overall quality of evidence is moderate. Further trials focusing on microbiological outcomes are needed to assess the efficacy of CHX in reducing the load of periodontal pathogens.

## Data Availability

The datasets used and/or analyzed during the current study are available from the corresponding author on reasonable request.

## Abbreviations

SRP: Scaling and root planing
CAL: Clinical attachment level
PPD: Probing pocket depth
CHX: Chlorhexidine
